# Is There Any Relationship between Plasma 25-Hydroxyvitamin D_3_, Adipokine Profiles and Excessive Body Weight in Type 2 Diabetic Patients?

**DOI:** 10.3390/ijerph15010019

**Published:** 2017-12-23

**Authors:** Joanna Kocot, Piotr Dziemidok, Małgorzata Kiełczykowska, Jacek Kurzepa, Grzegorz Szcześniak, Irena Musik

**Affiliations:** 1 Chair and Department of Medical Chemistry, Medical University of Lublin, Chodźki 4A, 20-093 Lublin, Poland; malgorzata.kielczykowska@umlub.pl (M.K.); jacek.kurzepa@umlub.pl (J.K.); irena.musik@umlub.pl (I.M.); 2 Diabetology Ward, Institute of Rural Health, Jaczewskiego 2, 20-090 Lublin, Poland; piotr.dziemidok@op.pl (P.D.); grzeszcze@poczta.wp.pl (G.S.); 3 Institute of Public Health, Pope John Paul II State School of Higher Education, Sidorska 95/97, 21-500 Biała Podlaska, Poland

**Keywords:** vitamin D, adipokines, type 2 diabetes, obesity

## Abstract

A growing interest in the role of vitamin D in metabolic diseases led us to study the relationships between 25-hydroxyvitamin D_3_ (25(OH)D_3_) and the profiles of selected adipokines in type 2 diabetic (T2DM) patients. The study comprised 92 type 2 diabetics divided into quartiles regarding 25(OH)D_3_ concentration. Each group was divided into male and female subgroups. All the studied patients had their anthropometric and biochemical parameters determined. Plasma 25-hydroxyvitamin D_3_ concentration was determined by HPLC, while the selected adipokines (leptin, adiponectin, resistin and visfatin) by ELISA methods. The ratio of leptin to adiponectin (L/A) was calculated for all the patients. In 85.3% of diabetics a full (<20 ng/mL) or moderate (20–30 ng/mL) vitamin D deficit was found. Irrespective of sex, plasma leptin concentration decreased across increasing quartiles of 25(OH)D_3_ level. In women, 25(OH)D_3_ was negatively correlated with BMI, leptin level as well as L/A ratio, and positively with adiponectin concentration. In men, 25(OH)D_3_ was positively correlated with HDL and negatively with systolic blood pressure (SBP), leptin level and L/A ratio. Considering all the patients, there ocurred a significant negative correlation between 25(OH)D_3_ and SBP, BMI, WHR, TG, leptin and L/A ratio and positive ones between 25(OH)D_3_ and both adiponectin and HDL. The results of the study support the existence of the relationship among vitamin D, obesity and leptin in type 2 diabetic patients.

## 1. Introduction

According to the World Health Organization (WHO), diabetes is defined as a group of metabolic diseases characterized by hyperglycemia due to impaired secretion and/or insulin action [1]. The incidence of diabetes is still growing and WHO has acknowledged it as one of the epidemic diseases of XXI^st^ century [1,2]. 

In the last decades, it has been increasingly recognized that vitamin D not only affects bone metabolism but also interacts with many other tissues and impacts several metabolic pathways, including adipocyte metabolism [3]. It is well known that an increased body fat and obesity are associated with a decrease in circulating 25(OH)D level. Experimental studies suggest that vitamin D_3_ deficiency, concomitant with obesity, can be connected with enhanced risk of insulin resistance and an increase in incidence of type 2 diabetes (T2DM) [4,5,6,7]. This was also confirmed by the recent meta-analysis which showed that hypovitaminosis D was associated with an elevated risk of future diabetes in older people [8]. Moreover, it has been found that both pancreatic β cells and preadipocytes/adipocytes express vitamin D receptor (VDR), whereas 1,25(OH)_2_D is suggested to enhance insulin secretion [9,10]. However, it is impossible to make unequivocal conclusion on importance of vitamin D_3_ in T2DM since clinical studies have provided divergent results [11,12,13,14,15]. 

The exact biochemical processes linking vitamin D deficit with obesity and/or T2DM need to be elucidated by further investigation. One of the possible hypotheses assumes that vitamin D may influence production and/or secretion of adipokines, such as leptin, adiponectin, resistin and visfatin, which affects insulin secretion and/or insulin sensitivity of peripheral tissues. 

Leptin has a beneficial effect on lipid metabolism: inhibits lipogenesis and stimulates lipolysis as well as the oxidation of fatty acids. It has also been proven to exert antidiabetic action. On the other hand, chronic hyperleptinaemia is thought to contribute to the development of T2DM [16]. The main metabolic role of adiponectin is to increase insulin sensitivity by enhancing fatty acid oxidation and inhibiting the liver synthesis of glucose [17]. Furthermore, it is believed to reduce the production of TNF-α—a cytokine considered as a marker of insulin resistance [18]. Resistin is believed to be a proinflammatory adipokine and its increase indirectly affects inflammation intensification [19], leading subsequently to insulin resistance of peripheral tissues. Visfatin participates in the synthesis of nicotinamide adenine dinucleotide (NAD^+^), which is involved in the regulation of glucose homeostasis. It is suggested that aging and hypercaloric feeding compromise visfatin-mediated NAD^+^ biosynthesis and thereby may contribute to type 2 diabetes development [20]. Moreover, the ratio of leptin to adiponectin is suggested to be a useful index of insulin resistance and a good indicator of anti-diabetic therapy effectiveness [21,22] as well as a potential biomarker for atherosclerosis in obese T2DM [23].

Considering the fact that the underlining mechanism involved in the crosstalk between vitamin D status and T2DM course are not completely evidenced, the present study is aimed at evaluating plasma 25-hydroxyvitamin D_3_ concentration (a marker of vitamin D) and its relationship to profile of selected adipokines in type 2 diabetics with excessive body weight. 

## 2. Materials and Methods

### 2.1. Study Subjects

The study included patients suffering from type 2 diabetes hospitalized in the Diabetology Ward of Institute of Rural Health in Lublin during autumn and winter (to minimise an effect of season on studied parameters). Ninety-two type 2 diabetic patients (48 women and 44 men), aged 18–64 (48.63 ± 15.07) were enrolled. The study included the patients who declared in questionnaire that they were not on exercise program at least half a year. The patients were all treated with oral blood glucose lowering medication.

Insulin-independent diabetes was recognized basing on the previous medical documentation confirming diagnosis or on the basis of the diagnostic process including laboratory examinations according to criteria of European Association for the Study of Diabetes as well as Polish Diabetes Association recommendations 2012 (Diabetes Poland 2012). 

The exclusion criteria were: insulin dependent (type 1) diabetes, concomitant disturbances of liver and thyroid, renal insufficiency, chronic inflammatory diseases as well as age over 65 (as cutaneous vitamin D synthesis is reduced in ageing persons), excessive alcohol consumption and vitamin D supplementation. 

Anthropometric parameters such as age, body mass index (BMI), waist-hip ratio (WHR), systolic blood pressure (SBP), diastolic blood pressure (DBP) as well as biochemical parameters such as total cholesterol (TC), high-density lipoprotein (HDL), low-density lipoprotein (LDL), triglycerides (TG) and glycated haemoglobin (HbA1c) were determined for all the studied patients. 

All subjects were informed about the aim of the experiment and gave written consent for the participation in the present study. The study protocol was approved by Bioethical Board of Medical University of Lublin, acceptance KE-0254/100/2012. The study was done in accordance with the Declaration of Helsinki (2000) of the World Medical Association.

### 2.2. Experimental Design

All the type 2 diabetic patients, enrolled in this study, were divided into quartiles considering plasma 25-hydroxyvitamin D_3_ concentration. Sex was also taken into account. The classification is presented in Table 1.

### 2.3. Biological Material Preparation 

Vein blood samples were drawn from subjects after overnight fast into heparinized test tubes. Plasma was separated by centrifugation (1000× *g*, 15 min), divided into several portions (to avoid thawing-freezing cycle) and kept in −70 °C for further examination. In the obtained material concentrations of 25-hydroxyvitamin D_3_ (25(OH)D_3_—a marker of vitamin D level in organism) as well as selected adipokines (leptin, adiponectin, resistin and visfatin) were all determined.

### 2.4. Determination of Plasma 25(OH)D_3_

Extraction and determination of plasma 25-hydroxyvitamin D_3_ were performed using Reagent kit for HPLC analysis of 25-OH-Vitamin D_2_/D_3_ in serum/plasma as well as the lyophilized serum calibrators and controls (bi-level I + II) for 25(OH)D_3_ and 25(OH)D_2_ produced by Chromsystems Instruments & Chemicals GmbH (Munich, Germany). The samples were extracted using solid-phase extraction. An HPLC system (Gilson, Inc., Middleton, WI, USA) with UV/Vis detector was used for chromatographic analysis. The separation of the analytes was performed according to manufacturer’s instruction (mobile phase flow rate—0.7 mL/min, temperature—25 °C, injection volume—25 µL, time of analysis—approx. 12 min, wavelength—265 nm, retention time—4.2 min). Data collection and quantitation were performed by the help of GILSON TRILUTION software. The concentration of 25(OH)D_3_ was expressed in ng/mL.

### 2.5. Determination of Plasma Selected Adipokines

The plasma concentrations of the studied adipokines were determined by immunoenzymatic methods using Human Leptin Quantikine ELISA Kit (R&D Systems, Minneapolis, MN, USA), Human Total Adiponectin/Acrp30 Quantikine ELISA Kit (R&D Systems), Human Resistin Quantikine ELISA Kit (R&D Systems) as well as Human Nicotinamide phosphoribosyltransferase, NAmPRTase ELISA Kit (Wuhan EIAab Science, Wuhan, China) for leptin, adiponectin, resistin and visfatin, respectively. The concentrations were expressed in ng/mL for leptin, resistin and visfatin and in µg/mL for adiponectin. The ratio of leptin to adiponectin (L/A) was calculated for all the patients.

## 3. Statistics

Statistical analysis was performed using Statistica version 12.0 PL (StatSoft, Tulsa, OK, USA). Shapiro-Wilk test was used to verify conformity of variables distribution to hypothetical normal distribution. Evaluation of variance homogeneity was performed using Leven’s test. In case of variables distinguished by normal distribution and homogenous variances mean value as well as standard deviation were given. When lack of conformity to normal distribution or heterogeneous variances occurred, variables were described using median as well as quartile distribution. For variables of normal distribution and homogenous variances difference significances were determined using a one-way analysis of variance (ANOVA) followed by Tukey HSD test, otherwise Kruskal–Wallis one-way analysis of variance by ranks together with multiple comparison post hoc test was applied. To evaluate relationships among studied parameters Spearman’s rank correlation coefficient test was applied. Values were considered significant with *p* < 0.05.

## 4. Results

### 4.1. Anthropometric Indices and Biochemistry

Anthropometric indices such as age, BMI, WHR, SBP, DBP, and biochemical parameters such as TC, LDL, HDL and triglycerides as well as HbA1c were compared among quartiles of 25-hydroxyvitamin D_3_ status. A significant difference was obtained only in case of BMI values in women and SBP in men, as shown in Table 1.

### 4.2. 25(OH)D_3_ Concentration in Diabetic Patients and Controls

Overall, 85.3% of diabetic patients exhibited vitamin D deficiency (<20 ng/mL) or vitamin D insufficiency (20–30 ng/mL). In women vitamin D deficiency was observed in 58.3% of patients, vitamin D insufficiency in 27.1%, whereas optimal concentration (>30 ng/cm^3^) was noted only in 14.6% of subjects. In men vitamin D deficiency was shown in 61.3%, its insufficiency in 27.3%, whereas optimal level only in 11.4% of patients.

### 4.3. Adipokines’ Profile Across Quartiles of Vitamin D Concentration 

**Figure 1:**
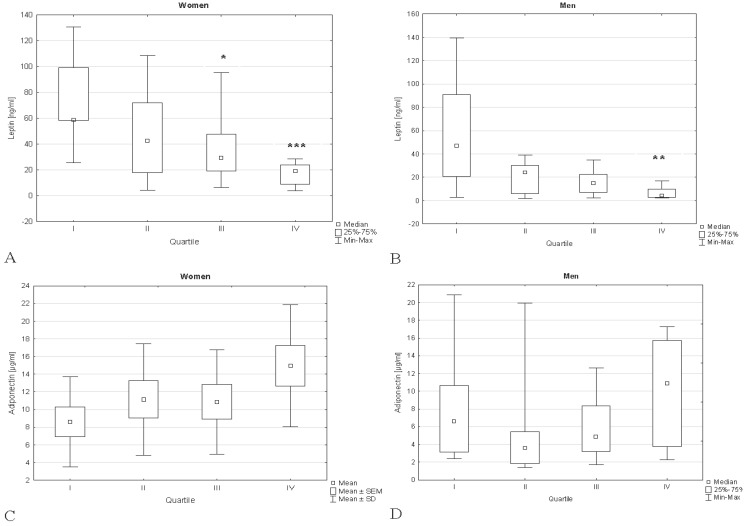

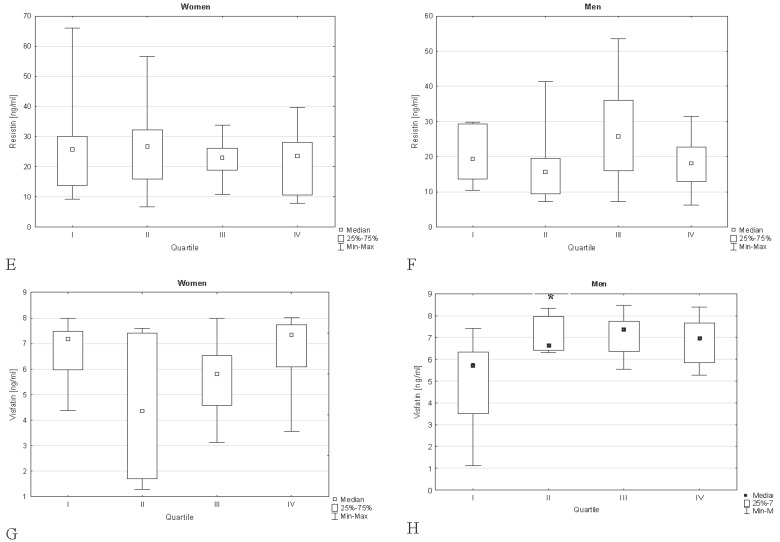
Concentration of adipokines in type 2 diabetics divided according to plasma 25(OH)D_3_ level. **A**: leptin in T2DM women; **B**: leptin in T2DM men; **C**: adiponectin in T2DM women; **D**: adiponectin in T2DM men; **E**: resistin in T2DM women; **F**: resistin in T2DM men; **G**: visfatin in T2DM women; **H**: visfatin inT2DM men. * *p* < 0.05 vs. quartile I, ** *p* < 0.01 vs. quartile I, *** *p* < 0.001 vs. quartile I. Guartile I: the lowest 25(OH)D_3_ concentration, quartile IV: the highest 25(OH)D_3_ concentration.

Irrespective of sex, a statistically significant decrease in leptin concentration was found in patients of quartile IV (the highest vitamin D) vs. quartile I (the lowest vitamin D). In women leptin was about threefold lower in quartile IV compared to I one, whereas in men over tenfold. Furthermore, in women leptin was also found to be significantly lower in quartile III vs. quartile I. A distinct tendency towards a leptin decrease along with a 25(OH)D_3_ increase was observed, both in women and in men. (Figure 1A,B). Irrespective of sex, the highest adiponectin concentration was found in quartile IV, but no statistically significant differences were observed. A slight tendency towards increasing of adiponectin level across 25-hydroksyvitamin D_3_ quartiles was found in women, whereas in men there was no such tendency (Figure 1C,D). 

No tendency towards changes of resistin level across 25-hydroksyvitamin D_3_ quartiles was observed (Figure 1E,F). In women, no significant differences between visfatin concentration in particular quartiles were shown. In men, visfatin was significantly increased in quartile II compared with quartile I. Both in women and men no tendency towards changes of visfatin concentration along with 25-hydroxyvitamin D_3_ concentration was displayed (Figure 1G,H).

### 4.4. Leptin/Adiponectin Ratio Across Quartiles of Vitamin D Concentration

**Figure 2:**
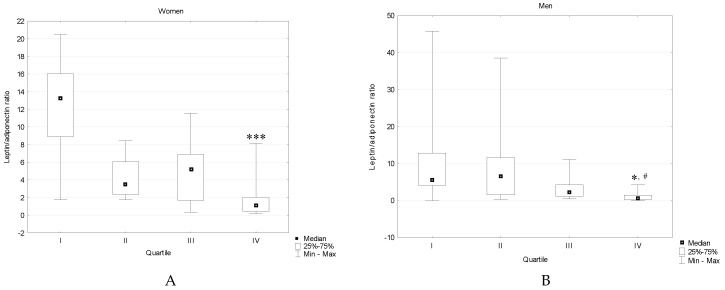
Leptin/adiponectin ratio in type 2 diabetic women (**A**) and men (**B**) divided according to plasma 25(OH)D_3_ level. * *p* < 0.05 vs. quartile I, *** *p* < 0.001 vs. quartile I, # *p* < 0.05 vs. quartile II.

In women, leptin/adiponectin ratio was decreased in quartile IV vs. quartile I, whereas in men vs. both quartile I and II. A slight tendency towards decreasing of leptin/adiponectin ratio across 25-hydroksyvitamin D_3_ quartiles (especially in women) was found (Figure 2). 

### 4.5. Correlations between 25-Hydroxyvitamin D_3_ and Anthropometric Indices, Biochemical Parameters as Well as Adipokines among Type 2 Diabetic Patients

In women, significant negative correlations between 25(OH)D_3_ level and BMI, leptin concentration as well as L/A ratio, and a significant positive correlation between 25(OH)D_3_ level and adiponectin were found. In men, 25(OH)D_3_ was strongly negatively correlated with SBP, leptin and L/A ratio, whereas between 25(OH)D_3_ and HDL a significant positive correlation was shown. Considering all diabetic patients, there were significant negative correlations between 25(OH)D_3_ and BMI, WHR, SBP, TG, leptin and leptin/adiponectin ratio, whereas positive ones between 25(OH)D_3_ and both adiponectin and HDL (Table 2). 

## 5. Discussion

Numerous studies found a high prevalence of vitamin D deficiency in patients with type 2 diabetes [6,24,25], which was also confirmed by the present study, as 85.3% of the studied patients exhibited moderate or severe deficiency of vitamin D (<30 ng/mL). The growing concern in vitamin D role in regulation of energetic metabolism and adipogenesis has been observed recently [26,27,28,29]. It has been prompted by in vitro studies which revealed direct effect of 1α,25-hydroxycholecalciferol on leptin and adiponectin secretion by adipocytes [30,31]. 

### 5.1. Leptin

In vitro studies considering the relationship between leptin and vitamin D have given inconclusive results. Kong et al. [32] found that 1α,25(OH)_2_D_3_ stimulated the expression of leptin by adipocytes isolated from mice. On the contrary, Menendez et al. [33] reported that vitamin D_3_ powerfully inhibited in vitro leptin secretion by human adipose tissue. Another study showed that the silencing of the genes encoding the vitamin D receptor and 1-α-hydroxylase reduced serum leptin and compensatory increased food intake in mice [34]. 

The current study displayed tendency towards leptin decrease along with 25(OH)D_3_ enhancement as well as the existence of a negative correlation between these parameters, both in women and men. Although in the available literature few studies concerning this relationship in humans can be found, the reported outcomes are consistent with those obtained in the present experiment. Amir et al. [35] observed a slight, negative correlation between 25(OH)D and leptin in women with breast cancer incidence risk. Figuiredo-Dias et al. [36] showed a negative correlation between 25(OH)D and leptin in patients with chronic kidney disease (31% of subjects suffered with type 2 diabetes). Grethen et al. [37] found a negative correlation between these parameters in obese women. It was also shown that administration of vitamin D led to decline in serum leptin level in end-stage renal disease patients on hemodialysis [38]. On the other hand, vitamin D supplementation was reported to significantly increase serum leptin in diabetic patients [26]. Ghavamzadeh et al. [39], in turn, showed that 14-week-supplementation with vitamin D had no effect on body weight, BMI value and amount of adipose tissue in insulin-independent diabetics. However, the observed twofold increase in 25(OH)D was connected with approximately twofold enhancement of leptin.

Maggi et al. [26] suggested that vitamin D, being a fat-soluble one, was accumulated in adipocytes which subsequently prompted leptin secretion. The accumulation of vitamin D in adipocytes may reduce its amount in the circulation that can be presented to the liver for 25-hydroxylation. The results of the current study seem to confirm this assumption as vitamin D was negatively correlated with BMI and WHR. Furthermore, regardless of the source, low circulating 1,25(OH)_2_D level could reduce circulating calcium level and induce secondary hyperparathyroidism, which stimulates lipogenesis and fat storage. This leads to an increased leptin secretion by adipocytes, which in turn stimulates fibroblast growth factor 23 expression in osteoblasts. This factor inhibits the activity of 1α-hydroxylase in the kidney/adipocytes, thereby reducing the synthesis of 1α,25 (OH)_2_D_3_, which further intensifies vitamin D deficiency [40]. On the contrary, the inverse correlation between plasma vitamin D and leptin level may indicate that vitamin D inhibits leptin secretion in vivo. The significance of the connections between leptin and vitamin D remains to be clarified.

Taken together, the facts presented above seems to indicate that it is possible that both factors can affect plasma leptin concentration separately, but coexistence of obesity and low 25(OH)D_3_ concentration intensify this effect. 

### 5.2. Adiponectin 

The existence of relationships between vitamin D and adiponectin under in vitro conditions was found [30,31]. According to Walker et al. [31], addition of 1α,25(OH)_2_D_3_ to growth medium induced adiponectin synthesis/secretion in mice cell line 3T31-L1. Moreover, Feng et al. [41] revealed 1α,25(OH)_2_D-induced reduction in the expression of TNFα mRNA—a proinflammatory cytokine, one of main factors inhibiting adiponectin synthesis. As both vitamin D and adiponectin are believed to possess anti-inflammatory properties, the existence of any relationships between these parameters under in vivo conditions seems to be possible. However, the outcomes of the current study did not confirm that assumption unequivocally. A slight tendency towards increasing of adiponectin level across 25-hydroksyvitamin D_3_ quartiles was found only in women. Considering all patients, 25(OH)D_3_ was positively correlated with adiponectin. However, when the patients were divided into sex subgroups, no such correlation was observed in men. Similarly, previous studies have yielded equivocal results [13,40,41,42,43,44,45]. It could result from the fact that the correlation between 25(OH)D_3_ and adiponectin depended on BMI or that 25(OH)D affected adiponectin level only to a small extent. This seems to be confirmed by the study of Breslavsky et al. [46], who showed that significant increase in 25(OH)D concentration following 12-month vitamin D supplementation was associated with a marginal enhancement of adiponectin level.

### 5.3. Resistin

The current study did not reveal any relationships between 25(OH)D_3_ and resistin. These results are consistent with those reported by other scientists. Al-Daghri et al. [42] did not found any correlation between resistin and 25(OH)D in type 2 diabetics. Another study [47], performed on obese and normal-weight-children, also showed no correlation between these parameters. Parikh et al. [48] in turn did not observe any relationships between 25(OH)D_3_ and resistin in healthy US teenagers. Stepien et al. [49] reported no influence of three-week-supplementation with vitamin D on resistin in healthy men. 

### 5.4. Visfatin

Similarly, as in the case of resistin, the present study showed no relationships between visfatin and vitamin D. In the literature data, there are only few studies investigating this relationship and their results were quite opposite. Zhang et al. [50] observed a visfatin decrease in women supplemented with 1α,25(OH)_2_D_3_ as well as a slight negative correlation between 25(OH)D_3_ and visfatin. The results obtained by Kiyak et al. [51] showed a negative correlation between vitamin D levels and visfatin in patients with polycystic ovary syndrome. The lack of any correlations between visfatin and vitamin D, observed in our study, could result from the fact that most subjects displayed vitamin D insufficiency/deficit. It may be possible that higher vitamin D concentration is required for affecting visfatin level.

### 5.5. Leptin/Adiponectin Ratio

We also evaluated leptin and adiponectin ratio since it has been believed to be a better determinant of insulin resistance and metabolic complications than either leptin or adiponectin alone. It was shown that higher 25-hydroxyvitamin D_3_ concentration was associated with decreased L/A ratio. To the best of our knowledge in the literature data there are only few studies investigating this relationship and their results are in line with ours. Belenchia et al. [13] found that after six months of vitamin D supplementation at a dose of 4000 IU/day obese adolescents showed significantly reduced L/A ratio, whereas Breslavsky et al. [46] revealed that 12-month, 1000 U/day vitamin D supplementation was associated with a decrease in L/A ratio in diabetic patients with the Hp 2-2 phenotype. Savastano et al. [52] in turn observed an inverse correlation between 25(OH)D and L/A ratio in women with polycystic ovary syndrome.

Our study has certain limitations. Firstly, this concerns a wide age range of the studied group. Although, there was no correlation in our study, we could not rule out the hypotheses that age could affect the studied parameters. It is well known that the ability of our body to produce vitamin D is decreasing with age. Furthermore, the association between age and adipokines’ levels it is not unambiguous. For example, a cross-sectional study carried out among 226 apparently healthy subjects showed that serum leptin level was not correlated with age in men, whereas in female a significant positive correlation was found [53]. In another study included 147 patients with T2DM no significant correlation between adiponectin and any clinical parameters, including age, was found [54], whereas Tingelstad et al. [55] in the study including 331 members of the regular Canadian Armed Forces showed that adiponectin was increasing with increasing age. Secondly, the physical activity was assessed based on personal questionnaire only. Thirdly, vitamin D status was estimated by measuring the level of plasma 25(OH)D_3_. It did not include vitamin D or its metabolites in peripheral tissues, such as adipose tissue and skeletal muscle.

## 6. Conclusions

We found an insufficiency of vitamin D in a large majority of selected type 2 diabetic patients. The results of the study support the existence of relationship among vitamin D, obesity and leptin in type 2 diabetic patients, which may indicate the role of leptin as a link between vitamin D status and type 2 diabetes. However, the future studies are needed to prove causality and directionality of these associations. 

## Figures and Tables

**Table 1:** The general characteristics of type 2 diabetic patients according to 25(OH)D_3_ status.

**Women**					
**Variable**	**Quartile I** **(*n* = 12)**	**Quartile II** **(*n* = 12)**	**Quartile III** **(*n* = 12)**	**Quartile IV** **(*n* = 12)**	**Total** **(*n* = 48)**
25(OH)D_3_ (ng/mL)	<10.87	10.88–19.41	19.42–25.81	>25.82	
Age	59.00 (56.00–63.00)	53.50 (33.00–61.50)	30.00 (26.00–43.00)	57.00 (52.00–61.00)	55.00 (30.00–62.00)
BMI (kg/m^2^)	32.33 (29.36–38.69)	24.30 (23.13–30.05)	23.99 (22.23–25.68) a ^(K)^	25.36 (23.44–30.80)	25.36 (23.18–32.36)
WHR	0.94 ± 0.09	0.84 ± 0.08	0.84 ± 0.09	0.89 ± 0.09	0.88 ± 0.09
SBP (mmHg)	134.42 ± 20.72	141.72 ± 22.90	136.23 ± 22.10	145.08 ± 13.66	139.25 ± 19.98
DBP (mmHg)	74.67 ± 11.77	77.73 ± 11.69	83.92 ± 12.51	78.00 ± 7.89	78.71 ± 11.32
HbA1c (%)	8.34 ± 2.33	7.09 ± 2.79	6.89 ± 2.00	8.30 ± 2.33	7.71 ± 2.37
TC (mg/dL)	172.63 ± 60.97	187.17 ± 39.83	178.62 ± 27.69	185.00 ± 28.97	180.98 ± 39.83
TG (mg/dL)	135.10 ± 56.08	111.25 ± 46.89	89.46 ± 38.43	128.91 ± 119.43	114.50 ± 71.129
HDL (mg/dL)	57.00 ± 17.02	61.17 ± 8.84	68.00 ± 16.13	69.75 ± 20.58	64.21 ± 16.49
LDL (mg/dL)	91.09 ± 43.09	105.63 ± 32.13	92.67 ± 27.71	91.63 ± 14.29	95.29 ± 30.28
**Men**					
**Variable**	**Quartile I** **(*n* = 11)**	**Quartile II** **(*n* = 11)**	**Quartile III** **(*n* = 11)**	**Quartile IV** **(*n* = 11)**	**Total** **(*n* = 44)**
25(OH)D_3_ (ng/mL)	<12.09	12.10–17.03	17.04–2.29	>22.30	
Age	45.41 ± 15.76	50.63 ± 14.73	47.75 ± 13.61	43.25 ± 13.74	46.68 ± 14.27
BMI (kg/m^2^)	34.01 ± 14.03	33.36 ± 9.36	30.81 ± 6.76	24.49 ± 3.59	30.61 ± 9.73
WHR	0.99 ± 0.10	1.00 ± 0.11	0.98 ± 0.08	0.90 ± 0.04	0.97 ± 0.09
SBP (mmHg)	150.50 ± 27.54	145.54 ± 32.81	137.27 ± 25.63	122.58 ± 13.95 *, # ^(T)^	138.86 ± 27.14
DBP (mmHg)	87.91 ± 22.85	84.63 ± 15.56	87.00 ± 11.05	75.41 ± 10.05	83.65 ± 16.10
HbA1c (%)	7.79 ± 2.57	7.28 ± 1.62	7.01 ± 2.19	6.26 ± 1.79	7.07 ± 2.09
TC (mg/dL)	171.58 ± 50.41	181.60 ± 45.2	204.83 ± 45.5	173.08 ± 44.29	182.82 ± 46.97
TG (mg/dL)	171.00 (88.00–222.00)	111.00 (98.00–128.00)	144.00 (103.00–248.00)	103.00 (94.00–132.00)	112.00 (93.00–208.00)
HDL (mg/dL)	40.50 (33.00–48.00)	49.50 (38.00–61.00)	48.50 (41.00–55.50)	63.00 (41.00–66.50)	46.50 (39.00–61.00)
LDL (mg/dL)	99.80 (93.40–122.60)	107.66 (98.00–131.20)	129.20 (106.60–138.00)	87.40 (54.10–111.10)	100.80 (87.40–129.80)

BMI: body mass index, WHR: waist-hip ratio, SBP: systolic blood pressure, DBP: diastolic blood pressure, HbA1c: glycated hemoglobin, TC: total cholesterol, TG: triglycerides, HDL: high-density lipoprotein, LDL: low-density lipoprotein. Normally distributed variables were expressed as mean ± SD, non-normal distributed ones as median and quartiles. * *p* = 0.03: Q4 vs. Q 1; # *p* = 0.02: Q4 vs. Q2; a *p* = 0.05: Q3 vs. Q1. ^T^ Tukey’s post-hoc test; ^K^ Kruskal-Wallis one way analysis of variance.

**Table 2:** Correlations between 25-hydroxyvitamin D_3_ and anthropometric indices, biochemical parameters as well as adipokines among type 2 diabetic patients.

25(OH)D_3_ (ng/mL)						
	Women (*n* = 48)		Men (*n* = 44)		Total (*n* = 92)	
	*r*	*p*	*r*	*p*	*r*	*p*
Age	−0.21	0.19	−0.12	0.47	−0.16	0.17
BMI (kg/m^2^)	−0.38	0.015	−0.30	0.07	−0.36	0.001
WHR	−0.30	0.06	−0.30	0.06	−0.33	0.003
SBP (mmHg)	−0.019	0.91	−0.51	0.04	−0.43	0.0003
DBP (mmHg)	−0.04	0.83	−0.02	0.94	−0.21	0.09
HbA1c (%)	0.18	0.40	−0.01	0.96	−0.08	0.51
TC (mg/dL)	0.16	0.34	0.02	0.92	0.09	0.43
TG (mg/dL)	0.30	0.08	−0.19	0.29	−0.26	0.03
HDL (mg/dL)	0.243	0.16	0.35	0.03	0.30	0.01
LDL (mg/dL)	0.06	0.71	0.06	0.72	0.02	0.84
Leptin (ng/mL)	−0.65	0.000007	−0.53	0.0007	−0.52	0.000001
Adiponectin (µg/mL)	0.33	0.044	0.23	0.17	0.26	0.02
L/A ratio	−0.63	0.00005	−0.57	0.0002	−0.54	0.000002
Resistin (µg/mL)	−0.11	0.49	0.12	0.47	0.01	0.92
Visfatin (µg/mL)	0.03	0.90	0.30	0.07	0.10	0.42

BMI: body mass index, WHR: waist-hip ratio, SBP: systolic blood pressure, DBP: diastolic blood pressure, HbA1c: glycated hemoglobin, TC: total cholesterol, TG: triglycerides, HDL: high-density lipoprotein, LDL: low-density lipoprotein. To evaluate relationships among studied parameters Spearman’s rank correlation coefficient test was applied.
